# The Emergence of Carbapenem- and Colistin-Resistant Enterobacteria in Senegal

**DOI:** 10.3390/pathogens12080974

**Published:** 2023-07-26

**Authors:** Habibou Sarr, Aissatou Ahmet Niang, Amadou Diop, Oleg Mediannikov, Hanane Zerrouki, Seydina M. Diene, Seynabou Lo, Mouhamadou Lamine Dia, Ahmad Iyane Sow, Florence Fenollar, Jean-Marc Rolain, Linda Hadjadj

**Affiliations:** 1 UFR des Sciences de la Santé, Université Assane Seck de Ziguinchor, Ziguinchor BP 523, Senegal; habibousarr10@gmail.com; 2 Unité de Bactériologie, Hôpital de la Paix de Ziguinchor, Ziguinchor BP 523, Senegal; 3 Faculté de Médecine et de Pharmacie, MEPHI IRD, APHM, Aix Marseille Université, 13005 Marseille, France; olegusss1@gmail.com (O.M.); zerrouki_hanane@hotmail.com (H.Z.); seydina.m.ddiene@gmail.com (S.M.D.); jean-marc.rolain@univ-amu.fr (J.-M.R.); 4 IHU Méditerranée Infection, 13005 Marseille, France; florence.fenollar@univ-amu.fr; 5 Faculté de Médecine, Pharmacie et Odonto-Stomatologie, Université Cheikh-Anta-Diop, Dakar BP 5005, Senegal; niangaisatou@yahoo.fr (A.A.N.); amadoudioplaba@yahoo.fr (A.D.); laminedia2004@gmail.com (M.L.D.); profisow3@gmail.com (A.I.S.); 6 UFR des Sciences de la Santé, Université Gaston Berger, Saint Louis BP 234, Senegal; zeynaby78@hotmail.fr; 7 VITROME, IRD, APHM, SSA, Aix Marseille Université, 13005 Marseille, France

**Keywords:** enterobacteria, carbapenemase, colistin

## Abstract

Antibiotic resistance is a public health problem. The emergence of carbapenemase-producing Enterobacterales (CPE) infections is a concern, particularly in Senegal. (1) Methods: Between January 2019 and July 2022, 240 isolates of enterobacteria resistant to third-generation cephalosporins and imipenem from biological samples from Fann Hospital (Dakar) and Hôpital Paix (Ziguinchor) were selected. The isolates were identified by MALDI-TOF mass spectrometry, and susceptibility tests were performed by the disk diffusion method. Antibiotic-resistance genes for class A beta-lactamases, carbapenemases, and plasmid resistance to colistin resistance (*mcr-1–8*) were screened by RT-PCR. (2) Results: The 240 enterobacteria were composed of: *Escherichia coli* (60.83%), *Klebsiella pneumoniae* (21.67%), *Enterobacter cloacae* (13.75%), *Citrobacter freundii* (2.08%), *Serratia marcescens* (0.83%), *Klebsiella aerogenes* (0.42%), and *Proteus mirabilis* (0.42%). Class A beta-lactamase genes were found in 229 isolates (70.41% *bla*_TEM_, 37.5% *bla*_SHV_, 83.75% *bla*_CTX-A_, and 0.42% *bla*_CTX-B_). The carbapenemase genes *bla*_OXA-48_ and *bla*_NDM_ were found in 25 isolates, including 14 isolates with *bla*_OXA-48_, 13 isolates with *bla*_NDM_, and 2 isolates with both genes simultaneously. The *mcr-8* gene was found in one isolate of *E. cloacae*. (3) Conclusions: The epidemiology of antibiotic-resistance genes in enterobacteria in Senegal shows the emergence of CPEs. This phenomenon is worrying, and rigorous surveillance is necessary to avoid further spread.

## 1. Introduction

Antibiotic resistance in enterobacterales is a public health problem that represents a threat to humans, animals, and the environment [1]. Multidrug-resistant enterobacterales infections result in longer hospital stays, higher patient care costs, and increased mortality [2]. Some of these infections are of nosocomial origin, thus complicating patient management [3,4]. Bacteria such as ESBL-producing *Enterobacteriaceae*, methicillin-resistant *Staphylococcus aureus*, and *Pseudomonas aeruginosa* are frequently isolated in hospital-acquired infections [5]. Antimicrobial resistance is one of the major health issues of this century and has a global impact on human health [6]. The global spread of antibiotic resistance and its mechanisms are constantly evolving and are often linked to countries’ antibiotics strategies [6,7]. Limited data are available to assess the level of antimicrobial resistance currently observed in developing countries (DCs), particularly in West Africa [8,9,10]. For instance, in Senegal, antibiotics are often prescribed empirically [11]. Overuse of third-generation cephalosporins (C3G) has favored the emergence of resistance toward these antibiotics [12,13]. As a result, carbapenems have been used since 2008 to combat ESBL-producing *Enterobacteriaceae* [14,15,16].

Although, colistin is mainly used in veterinary medicine [17], it has become increasingly used in some countries as a last-line therapeutic treatment against carbapenem-resistant isolates [18]. The introduction of these antibiotics into the clinical setting has led to the appearance and emergence of bacteria which are resistant to them [10,17,19].

The main mechanism of beta-lactam resistance in enterobacterales is enzymatic inactivation caused by proteins, known as beta-lactamases, encoded by antibiotic resistance genes [20]. The widespread of ESBL genes across a wide variety of species is mainly due to the presence of these genes on plasmids that facilitate their dissemination [21]. This also applies to the spread of carbapenemases as well as the plasmid-mediated colistin resistance genes “*mcr*”. Since 2009, a transferable colistin resistance mechanism (plasmid-mediated *mcr* gene) has been described worldwide [22,23]. Colistin resistance can also be chromosomal and is mainly mediated in *Enterobacteriaceae* via modification of the bacterial outer membrane by alteration of lipopolysaccharide chains (LPSs) [24]. This resistance also involves the secondary resistome, as inactivation of a gene not essential to bacterial multiplication and not involved in colistin resistance (dedA) restores its sensitivity [25,26]. In Senegal and Benin, TEM, SHV, OXA-1 and CTX-M-15 beta-lactamases are well disseminated [27,28,29]. Available clinical studies on the epidemiology of Carbapenemase-producing *Enterobacteriaceae* (CPE) in West and East Africa show the presence of carbapenemase genes in Cameroon (*bla*_NDM-4_), Kenya (*bla*_NDM-1_), Sierra Leone (*bla*_VIM_ and *bla*_DIM-1_), Senegal (*bla*_OXA-48_), and Tanzania (*bla*_KPC_, *bla*_IMP_, *bla*_OXA-48_, *bla*_VIM,_ and *bla*_NDM_) [14]. OXA-48 is the major carbapenemase produced by enterobacteria and the most common enzyme that has emerged in all Mediterranean countries as well as in Africa [30,31]. The first carbapenemase gene described in Senegal was OXA-48 in 2008, but there are probably some that predate this, as few regular epidemiological studies have taken place in Senegal [15,32]. Recently, the emergence of New Delhi metallo-β-lactamase (NDM-type) carbapenemases in Senegal and in the rest of Africa among *Acinetobacter baumannii* isolates has been reported [14,33].

The objective of this study was to update the epidemiology of class A beta-lactamases, carbapenemases, and plasmid-mediated colistin resistance in enterobacterales in two remote regions of Senegal: Dakar (west of Senegal) and Ziguinchor (south of Senegal). To do so, we performed a phenotypic and molecular study of antibiotic resistance and determined the genes associated with this resistance.

## 2. Materials and Methods

### 2.1. Selection of Isolates

Between January 2019 and July 2022, isolates of enterobacteria at Fann Hospital (Dakar, western Senegal) and Paix Hospital (Ziguinchor, southern Senegal) were selected. The selection was based on the results of an antibiogram performed on Mueller–Hinton agar with a champagne cork image and resistance to C3G (ceftriaxone, ceftazidime) and/or to imipenem. The selected enterobacteria were isolated from clinical samples received from the diagnostic laboratory as part of routine bacteriological testing. These samples came from patients who were hospitalized or who presented for a bacteriological check-up. At Fann Hospital, the samples were from the neurology, neurosurgery, infectious diseases, and otorhinolaryngology departments, whereas at Paix Hospital, the departments involved were urology, infectious diseases, otorhinolaryngology, and pediatrics.

### 2.2. Bacteriological Analyses

The isolates were sent for analysis to the IHU-Méditérranée Infection research laboratory in Marseille, France. Bacterial identification was performed using matrix-assisted laser desorption ionization-time of flight (MALDI-TOF) mass spectrometry (Bruker Daltonik, Bremen, Germany) [34]. Antibiotic susceptibility testing (AST) was conducted using the disk diffusion method, following the EUCAST 2022 recommendations [35]. *Escherichia coli* ATCC 25922 was used as a quality control for disk diffusion AST. When resistance was observed using the disk diffusion method, minimum inhibitory concentrations (MICs) of imipenem and ertapenem were determined using the E-test method (BioMérieux, Marcy l’Etoile, France), while the colistin MIC was determined using the UMIC microdilution method (Biocentric, Bandol, France). The β-CARBA test (Biorad, Hercules, CA, USA) was performed to detect carbapenemase activity in selected isolates. Sixteen antibiotics (i2A^®^, Montpellier, France) were used for AST; these included: amoxicillin(20 µg), amoxicillin/clavulanic acid (20/10 µg), cefepime (30 µg), piperacillin/tazobactam (36 µg), mecillinam (10 µg), ceftriaxone (30 µg), ertapenem (10 µg), imipenem (10 µg), fosfomycin (200 µg), furan (100 µg), trimethoprim/sulfamethoxazole (25 µg), amikacin (30 µg), ciprofloxacin (5 µg), tetracycline (30 µg), and gentamicin (10 µg).

### 2.3. Molecular Analysis

Bacterial DNA was extracted by heat shock [36]. Genes responsible for class A beta-lactamase production: *bla*_TEM_, *bla*_SHV_, *bla*_CTX-A_ (*bla*_CTX-M-1_ and *bla*_CTX-M-9_ group), and *bla*_CTX-B_ (*bla*_CTX-M-2_ and *bla*_CTX-M8/25_ group) as well as those encoding carbapenemases (*bla*_OXA-23_, *bla*_OXA-24_, *bla*_OXA-48_, *bla*_OXA-58_, *bla*_KPC_, *bla*_NDM_, *bla*_VIM_, and *bla*_IMP_) were screened in all isolates [37,38]; this is in addition to the plasmid-mediated colistin resistance genes *mcr-1*, *mcr-2*, *mcr-3*, *mcr-4*, *mcr-5*, and *mcr-8* [39,40,41,42]. Screening of resistance genes was conducted using the real-time PCR (using CFX96, C1000 Thermal Cycler, Bio-Rad, Hercules, CA, USA). This is except for *bla*_IMP_, which was tested by the standard PCR. The RT-PCR assay conditions were as follows: 50 °C for 2 min; 95 °C for 15 min; 95 °C for 1 s; 60 °C for 30 s × 35 cycles; and 45 °C for 30 s. Standard PCRs (using 2720 Thermal Cycler, Applied Biosystems, Waltham, MA, USA), on the other hand, were performed as follows: 50 °C for 2 min; 96 °C for 15 min; 94 °C for 1 min; 55 °C for 50 s; 72 °C for 2 min × 35 cycles; and 72 °C for 7 min; 15 °C.

## 3. Results

### 3.1. Distribution of Samples and Isolates

The following samples were analyzed at the laboratory of Paix Hospital in Ziguinchor: urine (28.33%), pus (5.42%), blood culture (1.67%), and sputum (0.42%). In the laboratory of Fann Hospital in Dakar, the following samples were analyzed: urine (33.75%), pus (17.5%), blood culture (3.75%), vaginal secretions (5.0%), puncture fluid (3.33%), and sputum (0.83%). Overall, 240 isolates of enterobacteria were isolated from various biological samples: 86 from Ziguinchor and 154 from Dakar (Table 1). The distribution of isolates by year is detailed in Supplementary Table S1.

### 3.2. Antibiotic Resistance Phenotype

**Figure 1:**
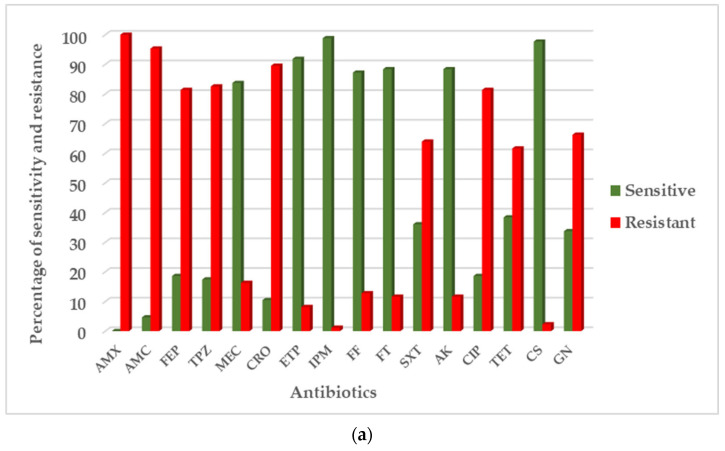

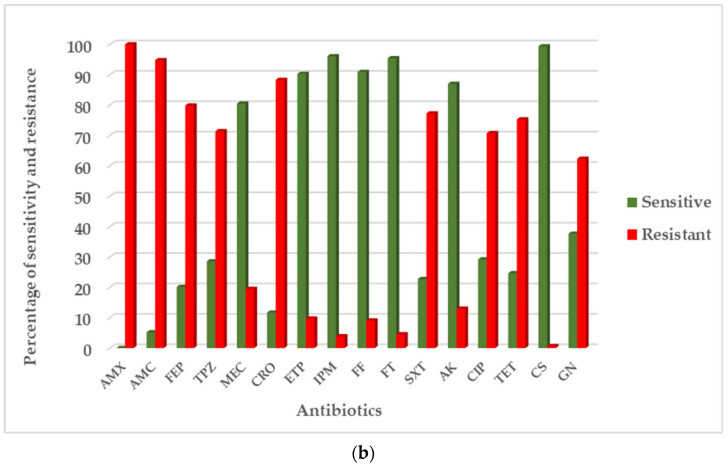
(**a**) Antibiotic resistance profile of enterobacteria in Ziguinchor. (**b**) Antibiotic resistance profile of enterobacteria in Dakar. AMX = amoxicillin, AMC = amoxicillin/clavulanic acid, FEP = cefepime, TPZ = piperacillin/tazobactam, MEC = mecillinam, CRO = ceftriaxone, ETP = ertapenem, IPM = imipenem, FF = fosfomycin, FT = furan, SXT = trimethoprim/sulfamethoxazole, AK = amikacin, CIP = ciprofloxacin, TET = tetracycline, CS = colistin, and GN = gentamicin.

For all enterobacteria tested, the resistance phenotypes showed 100% resistance to amoxicillin, 95.8% resistance to amoxicillin/clavulanic acid, 75.8% resistance to cefepime, 75.4% resistance to piperacillin/tazobactam, 90% resistance to ceftriaxone, 9.2% resistance to ertapenem, and 2.9% resistance to imipenem. On the other hand, in Ziguinchor, AST of the isolates revealed 100% resistance to amoxicillin, 95.3% resistance to amoxicillin/clavulanic acid, 81.4% resistance to cefepime, 82.6% resistance to piperacillin/tazobactam, 89.5% resistance to ceftriaxone, 8.1% resistance to ertapenem, and 1.2% resistance to imipenem (Figure 1a). In Dakar, the resistance phenotypes showed 100% resistance to amoxicillin, 94.8% resistance to amoxicillin/clavulanic acid, 79.9% resistance to cefepime, 71.4% to piperacillin/tazobactam, 88.3% resistance to ceftriaxone, 9.7% resistance to ertapenem, and 3.9% resistance to imipenem (Figure 1b). Antibiotic susceptibility profiles by bacterial species are detailed in Supplementary Table S2. We found two isolates (*E. cloacae* and *C. freundii*) resistant to both imipenem (MIC > 4 mg/L) and ertapenem (MIC > 0.5 mg/L). One isolate of *E. cloacae* was heteroresistant to colistin (MIC > 2 mg/L). The β-CARBA test was positive for all enterobacteria that were resistant to imipenem and/or ertapenem.

### 3.3. Distribution of Class A Beta-Lactamase Genes

**Figure 2:**
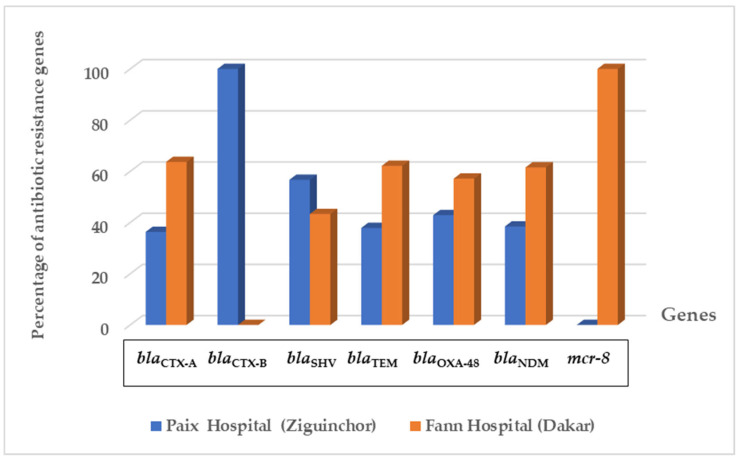
Distribution of antibiotic resistance genes in the two hospitals.

The class A beta-lactamase genes responsible were found in 229 isolates. The *bla*_CTX-A_, *bla*_CTX-B_, *bla*_SHV_, and *bla*_TEM_ genes were found in 83.75%, 0.42%, 37.5%, and 70.41% of enterobacteria isolates, respectively (Table 2). These genes were found in isolates from both hospitals (Figure 2). The *bla*_CTX-A_ gene was found in 84.2% of *E. coli*, 96.1% of *K. pneumoniae,* and 66.6% of *E. cloacae*. The *bla*_NDM_ gene was found in 3.4% of *E. coli*, 7.7% of *K. pneumoniae,* and 12.1% of *E. cloacae***.** The distribution of genes by year is detailed in Supplementary Table S1.

### 3.4. Carbapenem Resistance Genes

The *bla*_OXA-48_ and *bla*_NDM_ genes were found in 25 isolates (14 strains with *bla*_OXA-48_ and 13 strains with *bla*_NDM_) and with those two strains (*K. pneumoniae* and *E. cloacae*) which produced double carbapenemase. The *bla*_OXA-48_ gene was found in six isolates at Paix Hospital in Ziguinchor and in eight isolates at Fann Hospital in Dakar (Figure 2). The *bla*_NDM_ gene was found in five isolates at Paix Hospital and in eight isolates at Fann Hospital. The *bla*_OXA-48_ gene was mainly observed in *E. coli* (3.4%), *E. cloacae* (15.1%), *K. pneumoniae* (5.7%), and *C. freundii* (20%), while the *bla*_NDM_ gene was mainly detected in *E. coli* (3.4%), *E. cloacae* (12.1%), and *K. pneumoniae* (7.7%) (Table 2). The isolates with the *bla*_OXA-48_ and *bla*_NDM_ genes came from the neurology, neurosurgery, and infectious disease departments of Fann Hospital, while the isolates with the *bla*_OXA-48_ and *bla*_NDM_ genes came from the urology, infectious disease, and otorhinolaryngology departments of Paix Hospital. The *bla*_OXA-23_, *bla*_OXA-24_, *bla*_OXA-58_, *bla*_KPC_, *bla*_VIM_, and *bla*_IMP_ genes were not found in our isolates.

### 3.5. Plasmid Colistin Resistance Genes

The *mcr-8* gene was found in an isolate of *E. cloacae* isolated from a patient hospitalized in the infectious diseases department of Fann Hospital in Dakar (Figure 2, Table 2). None of our isolates harbored the *mcr-1*, *mcr-2*, *mcr-3*, *mcr-4*, or *mcr-5* genes.

## 4. Discussion

In Senegal, the epidemiology of antibiotic resistance genes in clinically isolated enterobacteria is poorly understood, and information is often outdated [27,32,43]. There is a pressing need, therefore, to strengthen and update this epidemiological data. Our study focused on a variety of enterobacteria which were resistant to C3G and/or imipenem. For all tested isolates, resistance to C3G and C4G was 88.3% and 79.9% in Dakar, respectively, and 89.5% and 81.4% in Ziguinchor, respectively. Across the country, *E. coli* resistance to ceftriaxone was 81.5%, and cefepime resistance was 75.2%. Class A beta-lactamase genes such as *bla*_TEM_ and/or *bla*_SHV_ were found in 66% of the isolates, whereas *bla*_CTX-A_ genes were found in 84% of the *E. coli* isolates. The expression of the *bla*_CTX-A_ genes confers high levels of resistance toward C3G (cefotaxime, ceftriaxone, and ceftazidime) and aztreonam [44]. A previous study carried out between 2009 and 2010 at Fann Hospital (Dakar, Senegal) reported the detection of *bla*_TEM_, *bla*_SHV_, *bla*_OXA-1_, and *bla*_CTX-M-15_ genes, with a predominance of the *bla*_CTX-M-15_ gene in *E. coli* isolates [27]. The prevalence of class A beta-lactamase genes found in our study (*bla*_TEM_, *bla*_SHV_, *bla*_CTX-A_, and *bla*_CTX-B_ in *E. coli*) is comparable to the results of the latter study and demonstrates the persistence and establishment of this antibiotic resistance genotype in this hospital over ten years. Similarly, in a study conducted in Benin, these same genotypes were found in enterobacterales, thus demonstrating their distribution in West Africa [28,29]. Apart from isolates of clinical origin, strains isolated from animals and the environment also harbored *bla*_CTX-M_ genes [45], suggesting a synergy of action between the three sectors (the “One Health” concept). Indeed, most ESBL genes are carried by plasmids and, thus, are easily transferable between different species of enterobacteria [21]. The *bla*_SHV_, *bla*_TEM_, *bla*_CTX-A_, and *bla*_CTX-B_ genes have also been described in other enterobacteria such as *K. pneumoniae* and *E. cloacae* isolates. Other studies carried out in Senegal found the *bla*_CTX-M-15_ gene in *Morganella morganii* and the *bla*_CTX-M_ group 1 and *bla*_CTX-M_ group 9 genes in other enterobacterial species (*E. coli*, *K. pneumoniae*, *E. cloacae*, and *C. koseri*) isolated from urine, thus demonstrating the diversity of clinical enterobacteria isolates carrying these antibiotic resistance genes [43,46]. The genotypic results in Senegal confirm the presence as well as the circulation of CTX-M ESBL genes in *E. coli* between the west and the south of the country. Our study therefore shows the evolution of ESBL epidemiology in this country. This is with the emergence of new ESBL-carrying isolates such as *C. freundii*, *E. aerogenes*, *P. mirabilis,* and *S. marcescens*, as well as the appearance of carbapenemases, as is the case in other parts of the world [47].

The carbapenemase genes (*bla*_OXA-48_ and *bla*_NDM_) were found in *E. coli*, *K. pneumoniae* and *E. cloacae* species, resulting in resistance to imipenem and/or ertapenem. This resistance is low in Dakar and Ziguinchor, where ertapenem resistance is only 9.7% and 8.1%, respectively, and imipenem resistance is 3.9% and 1.2%, respectively. Previous studies conducted in two Senegal hospitals, in Dakar, reported the detection of the *bla*_OXA-48_ gene in 11 isolates of enterobacteria between 2008 and 2009. On the other hand, in Saint Louis, *bla*_OXA-48_ was detected in 49 isolates of *K. pneumoniae* isolated from urine in 2016 [15,19,32]. *bla*_OXA-48_ is mainly found in *K. pneumoniae* and in *E. coli*, although other enterobacterial species can express this carbapenemase. In our study, in addition to these two species, *C. freundii* and *E. cloacae* isolates also harbored this carbapenemase gene. In the 14 isolates carrying the *bla*_OXA-48_ gene, we found resistance to ceftriaxone and cefepime and sensitivity to imipenem, and nine showed resistance to ertapenem. The OXA-48 gene has been described as the phantom threat, due to its discrete phenotype in the absence of co-resistance mechanisms [48]. In addition to *bla*_OXA-48_, we observed the *bla*_NDM_ gene in 13 isolates of enterobacteria, including three isolates of *K. pneumoniae*, three isolates of *E. cloacae,* and four isolates of *E. coli*. Both carbapenemase genes were found in isolates from the Paix (Ziguinchor) as well as Fann hospitals (Dakar), showing a homogeneous epidemiology between the west and the south of the country. The NDM enzyme is one of the most common carbapenemases in enterobacterales that is also observed in *A. baumannii* isolates [49,50,51]. In Senegal, NDM was first described in 2016 in an isolate of *A. baumannii,* and the literature has not yet reported its detection in enterobacteria [14,15]. Our results show that the NDM gene is present in both enterobacterial isolates isolated in western and southern Senegal. Elsewhere in Africa, carbapenemases are more commonly described in *Pseudomonas aeruginosa* and in *A. baumannii*, with the NDM enzyme being less frequently observed than OXA-48 [14]. The few available studies on the epidemiology of CPEs in West and East Africa report the identification of carbapenemases in Cameroon (NDM-4), Kenya (NDM-1), Sierra Leone (VIM and DIM-1), Senegal (OXA-48), and Tanzania (KPC, IMP, OXA-48, VIM, and NDM) [14].

Besides carbapenemase genes, colistin plasmid-mediated resistance genes deserve special attention. Indeed, colistin is much more widely used in veterinary medicine [17,52]. In human medicine, it represents a therapeutic option of last resort for the treatment of severe infections caused by multidrug-resistant enterobacteria [53,54,55]. In our study, we found an *E. cloacae* isolate with the *mcr-8* gene, which was sensitive to colistin (MIC = 0.25 mg/L) and another *E. cloacae* isolate which was heteroresistant to colistin. This heteroresistance is indeed natural in some isolates of *E. cloacae* [56]. The isolate with the *mcr-8* gene was isolated from a patient (a vegetable farmer) hospitalized in the infectious diseases department (Fann Hospital) for bacteremia. The *mcr-8* gene was first described in China in an isolate of *K. pneumoniae* of animal origin [57]. This gene is often detected in isolates of poultry meat origin in veterinary settings. In Senegal, a low prevalence of colistin resistance was observed in 93 *E. coli* (2.2%) isolated from poultry, with neither the *mcr-1* nor *mcr-2* gene being detected [17]. Furthermore, in Africa, *mcr* genes are rarely clinically described. In Algeria, the *mcr-8* gene was found in clinically isolated *K. pneumoniae* [41]. In France, the *mcr-1* gene has been described in an *E. cloacae* isolate [58]. In Senegal, resistance to colistin is rarely described clinically, as this molecule is not yet prescribed for use in humans. In fact, in countries with limited resources, such as Senegal, it is important to educate on and raise awareness of the correct use of antibiotics in order to combat the spread of antibiotic-resistant strains [59].

## 5. Conclusions

Although studies and epidemiological surveillance on antibiotic resistance remain limited in Senegal, this work has uncovered a new epidemiological aspect of antibiotic-resistant isolates in the country. There is a “persistence” of ESBL-producing *E. coli* isolates and a spread of ESBL as well as carbapenemase genes such as OXA-48 and NDM to other enterobacterial species. Even if colistin resistance appears to be anecdotal, it is important to monitor this emergence. In view of this, it has become of paramount importance to set up a regular surveillance system to prevent the large-scale dissemination of antibiotic-resistant bacteria, especially CPE.

## Figures and Tables

**Table 1:** Distribution of isolates per hospital.

Species	Number of Isolates (%)		Total (%)
	Paix Hospital (Ziguinchor)	Fann Hospital (Dakar)	
*Escherichia coli*	35 (14.58%)	111 (46.25)	146 (60.83%)
*Klebsiella pneumoniae*	29 (12.08%)	23 (9.59%)	52 (21.67%)
*Enterobacter cloacae*	16 (6.65%)	17 (7.08%)	33 (13.75%)
*Citrobacter freundii*	4 (1.66%)	1 (0.42%)	5 (2.08%)
*Klebsiella aerogenes*	1 (0.42%)	0 (0%)	1 (0.42%)
*Proteus mirabilis*	0 (0%)	1 (0.42%)	1 (0.42%)
*Serratia marcesens*	1 (0.42%)	1 (0.42%)	2 (0.84%)
Total (%)	86 (35.83%)	154 (64.17%)	240 (100%)

**Table 2:** Distribution of isolates according to antibiotic resistance genes.

Species	Number (%)	Antibiotic Resistance Genes						
		*bla*_CTX-A_	*bla*_CTX-B_	*bla*_SHV_	*bla*_TEM_	*bla*_OXA-48_	*bla*_NDM_	*mcr-8*
*Escherichia coli*	146 (60.83%)	123 (84.2%)	1 (0.6%)	26 (17.8%)	90 (61.6%)	5 (3.4%)	5 (3.4%)	0 (0%)
*Klebsiella pneumoniae*	52 (21.67%)	50 (96.1%)	0 (0%)	52 (100%)	46 (88.4%)	3 (5.7%)	4 (7.7%)	0 (0%)
*Enterobacter cloacae*	33 (13.75%)	22 (66.6%)	0 (0%)	10 (30.3%)	27 (81.8%)	5 (15.1%)	4 (12.1%)	1 (3.0%)
*Citrobacter freundii*	5 (2.08%)	4 (80%)	0 (0%)	1 (20%)	5 (100%)	1 (20%)	0 (0%)	0 (0%)
*Klebsiella aerogenes*	1 (0.42%)	0 (0%)	0 (0%)	0 (0%)	0 (0%)	0 (0%)	0 (0%)	0 (0%)
*Proteus mirabilis*	1 (0.42%)	1 (100%)	0 (0%)	0 (0%)	1 (100%)	0 (0%)	0 (0%)	0 (0%)
*Serratia marcescens*	2 (0.83%)	1 (50%)	0 (0%)	1 (50%)	0 (0%)	0 (0%)	0 (0%)	0 (0%)
Total (%)	240 (100%)	201 (83.75%)	1 (0.42%)	90 (37.5%)	169 (70.41%)	14 (5.83%)	13 (5.41%)	1 (0.42%)

## Data Availability

Not applicable.

## Supplementary Materials

The following supporting information can be downloaded at: https://www.mdpi.com/article/10.3390/pathogens12080974/s1. Table S1: Distribution of strains isolated and antibiotic resistance genes detected by year; Table S2: Distribution of antibiotic resistance by bacterial species.
