# Supplementary material for: The Emergence of Carbapenem- and Colistin-Resistant Enterobacteria in Senegal
Source: Pathogens. 2023 Jul 26;12(8):974. doi: 10.3390/pathogens12080974 (PMC10459028; doi:10.3390/pathogens12080974)
Supplement: Supplementary file 1 [file pathogens-12-00974-s001.zip › pathogens-2441708-supplementary.pdf]

pathogens

MDPI

Article

The Emergence of Carbapenem- and Colistin-Resistant Enterobacteria in Senegal

Habibou Sarr [1,2,3,4], Aissatou Ahmet Niang [5], Amadou Diop [5], Oleg Mediannikov [3,4], Hanane Zerrouki [3,4], Seydina M. Diene [3,4], Seynabou Lo [6], Mouhamadou Lamine Dia [5], Ahmad Iyane Sow [5], Florence Fenollar [4,7], Jean-Marc Rolain [3,4] and Linda Hadjadj [3,4,*]

[1] UFR des Sciences de la Santé, Université Assane Seck de Ziguinchor, Ziguinchor BP 523, Senegal; habibousarr10@gmail.com
[2] Unité de Bactériologie, Hôpital de la Paix de Ziguinchor, Ziguinchor BP 523, Senegal
[3] Faculté de Médecine et de Pharmacie, MEPHI IRD, APHM, Aix Marseille Université, 13005 Marseille, France; olegusss1@gmail.com (O.M.); zerrouki_hanane@hotmail.com (H.Z.); seydina.m.ddiene@gmail.com (S.M.D.); jean-marc.rolain@univ-amu.fr (J.-M.R.)
[4] IHU Méditerranée Infection, 13005 Marseille, France; florence.fenollar@univ-amu.fr
[5] Faculté de Médecine, Pharmacie et Odonto-Stomatologie, Université Cheikh-Anta-Diop, Dakar BP 5005, Senegal; niangaisatou@yahoo.fr (A.A.N.); amadoudioplaba@yahoo.fr (A.D.); laminedia2004@gmail.com (M.L.D.); profisow3@gmail.com (A.I.S.)
[6] UFR des Sciences de la Santé, Université Gaston Berger, Saint Louis BP 234, Senegal; zeynaby78@hotmail.fr
[7] VITROME, IRD, APHM, SSA, Aix Marseille Université, 13005 Marseille, France
* Correspondence: linda.hadjadj@univ-amu.fr; Tel.: +33-4-86-13-68-30

Citation: Sarr, H.; Niang, A.A.; Diop, A.; Mediannikov, O.; Zerrouki, H.; Diene, S.M.; Lo, S.; Dia, M.L.; Sow, A.I.; Fenollar, F.; et al. The Emergence of Carbapenem- and Colistin-Resistant Enterobacteria in Senegal. *Pathogens* **2023**, *12*, 974. https://doi.org/10.3390/pathogens12080974

Academic Editors: Carla Calia and Lawrence S. Young

Received: 24 May 2023
Revised: 19 July 2023
Accepted: 24 July 2023
Published: 26 July 2023

Copyright: © 2023 by the authors. Submitted for possible open access publication under the terms and conditions of the Creative Commons Attribution (CC BY) license (https://creativecommons.org/licenses/by/4.0/).

Supplementary Material

Table S1. Distribution of strains isolated, and antibiotic resistance genes detected by year.

		**Years**			
		**2019**	**2020**	**2021**	**2022**
**Bacterial Species**	*E. coli* (*n* = 146)	51 (34.9%)	47 (32.2%)	36 (24.7%)	12 (8.2%)
	K. pneumoniae (*n* = 52)	0 (0%)	5 (96%)	24 (46.2%)	23 (44.2%)
	E. cloacae (*n* = 33)	6 (18.2%)	10 (30.3%)	10 (30.3%)	7 (21.2%)
	C. freundii (*n* = 5)	0 (0%)	1 (20%)	2 (40%)	2 (40%)
	K. aerogenes (*n* = 1)	0 (0%)	0 (0%)	1 (100%)	0 (0%)
	P. mirabilis (*n* = 1)	0 (0%)	0 (0%)	0 (0%)	1 (100%)
	S. marcescens (*n* = 2)	1 (50%)	1 (50%)	0 (0%)	0 (0%)
**Genes**	bla_{CTX-A}	48 (23.9%)	47 (23.4%)	64 (31.8%)	42 (20.9%)
	bla_{CTX-B}	0 (0%)	1 (100%)	0 (0%)	0 (0%)
	bla_{SHV}	4 (4.4%)	16 (17.8%)	38 (42.2%)	32 (35.6%)
	bla_{TEM}	38 (22.5%)	46 (27.2%)	52 (30.8%)	33 (19.5%)
	bla_{OXA-48}	2* (14.3%)	2* (14.3%)	6* (42.9%)	4* (28.6%)
	bla_{NDM}	3* (23.1%)	0 (0%)	6* (46.2%)	4* (30.8%)
	mcr-8	0 (0%)	1 (100%)	0 (0%)	0 (0%)
**Hospitals**	Ziguinchor	0 (0%)	19 (22.1%)	47 (54.7%)	20 (23.3%)
	Dakar	58 (37.7%)	45 (29.2%)	26 (16.9%	25 (16.2%)

* Strains with a positive ß-CARBA test.

Table S2. Distribution of antibiotic resistance by bacterial species.

	**Bacterial Species**						
**Antibiotics**	***E. coli* (*n* = 146)**	***K. pneumoniae* (*n* = 52)**	***E. cloacae* (*n* = 33)**	***C. freundii* (*n* = 5)**	***K. aerogenes* (*n* = 1)**	***P. mirabilis* (*n* = 1)**	***S. marcescens* (*n* = 2)**
**AMX**	146 (100%)	52 (100%)	33 (100%)	5 (100%)	1 (100%)	1 (100%)	2 (100%)
**AMC**	139 (95.2%)	49 (94.2%)	33 (100%)	5 (100%)	1 (100%)	1 (100%)	2 (100%)
**FEP**	110 (75.3%)	50 (96.2%)	25 (75.8%)	4 (80%)	0 (0%)	0 (0%)	1 (50%)
**TPZ**	106 (72.6%)	47 (90.4%)	22 (66.7%)	4 (80%)	1 (100%)	1 (100%)	0 (0%)
**MEC**	21 (14.4%)	13 (25%)	6 (18.2%)	1 (20%)	0 (0%)	1 (100%)	2 (100%)
**CRO**	119 (81.5%)	51 (98.1%)	29 (87.9%)	4 (80%)	0 (0%)	1 (100%)	2 (100%)
	* 5 (1–4)	* 7 (0.75–6)	* 8 (0.74–24)	* 2 (2–12)	0	0	0

**ETP**	(3.4%)	(13.5%)	(24.2%)	(40%)	(0%)	(0%)	(0%)
**IPM**	* 1 (4) (0.7%)	* 2 (4) (3.8%)	* 2 (4–6) (6.1%)	* 1 (32) (20%)	0 (0%)	* 1 (3) (100%)	0 (0%)
**FF**	2 (1.4%)	12 (23.1%)	11 (33.3%)	0 (0%)	0 (0%)	0 (0%)	0 (0%)
**FT**	7 (4.8%)	5 (9.6%)	4 (12.1%)	0 (0%)	0 (0%)	0 (0%)	1 (50%)
**SXT**	111 (76%)	37 (71.2%)	22 (66.7%)	2 (40%)	0 (0%)	1 (100%)	1 (50%)
**AK**	21 (14.4%)	6 (11.5%)	2 (6.1%)	0 (0%)	0 (0%)	0 (0%)	1 (50%)
**CIP**	111 (76%)	39 (75%)	33 (100%)	5 (100%)	0 (0%)	1 (100%)	0 (0%)
**TET**	118 (80.8%)	27 (51.9%)	20 (60.6%)	2 (40%)	0 (0%)	1 (100%)	1 (50%)
**CS**	0 (0%)	0 (0%)	1 (>64) (3%)	0 (0%)	0 (0%)	1 (>64) (100%)	2 (>64) (100%)
**GN**	85 (58.2%)	42 (80.8%)	21 (63.6%)	3 (60%)	0 (0%)	1 (100%)	1 (50%)

AMX = Amoxicillin, AMC = Amoxicillin/Clavulanic Acid, FEP = Cefepime, TPZ = Piperacillin/Tazobactam, MEC = Mecillinam, CRO = Ceftriaxone, ETP = Ertapenem, IPM = Imipenem, FF = Fosfomycin, FT = Furan, SXT = Trimethoprim/Sulfamethoxazole, AK = Amikacin, CIP = Ciprofloxacin, TET = Tetracycline, CS = Colistin, GN = Gentamicin. * = MIC value in mg/L.
